# Editorial: Metabolically healthy and unhealthy obese children and adolescents, volume II

**DOI:** 10.3389/fendo.2022.1111060

**Published:** 2022-12-19

**Authors:** Bo Xi, Cristina Cadenas-Sanchez

**Affiliations:** ^1^ Department of Epidemiology, School of Public Health, Shandong University, Jinan, China; ^2^ Sport and Health University Research Institute (iMUDS), Department of Physical and Sports Education, Faculty of Sport Sciences, University of Granada, Granada, Spain; ^3^ Centro de Investigación Biomédica en Red Fisiopatología de la Obesidad y Nutrición (CIBERobn), Instituto de Salud Carlos III, Madrid, Spain

**Keywords:** metabolic phenotype, obesity, factors, adiposity, youth

## Introduction

The prevalence of obesity in youth is increasing globally. It is already known that obesity in youth is positively associated with metabolic risk factors such as high blood pressure, dyslipidemia, and high blood glucose. However, there are also some young people who are obese but have no metabolic risk factors, and this status is usually referred to as “metabolically healthy obesity” (MHO). 

Recent meta-analysis, including 23 prospective cohort studies of 4,492,723 participants, has shown that MHO is positively associated with the risk of cardiovascular disease when compared with metabolically healthy normal weight in adults (1). Evidence suggests that MHO tends to evolve into metabolically unhealthy obesity, thus leading to a higher risk of cardiovascular disease over time (2). There is also significant evidence to suggest that MHO is associated with high carotid intima-media thickness (3) and left ventricular hypertrophy (as observed by Genovesi et al. in this issue) in the pediatric population. Thus, it is important to know the accurate definition, current prevalence, and associated factors of MHO in youth.

## Definition of MHO

Currently, the definition of MHO in youth is controversial and heterogeneous, resulting in the prevalence estimates of MHO ranging from 3% to 80% (4). There are two definitions that are widely used in literature, including the modified National Cholesterol Education Program (NCEP) criteria (5) and the modified International Diabetes Federation (IDF) criteria (6), which are widely referred to for the definitions of metabolic syndrome in youth. The metabolic risk factors in both definitions include elevated blood pressure, elevated triglycerides, low high-density lipoprotein cholesterol, and elevated fasting blood glucose, although the cut-offs of the components are different. In this issue, Zong et al. call for the global unification of the definition of metabolic syndrome in youth for the direct comparison of prevalence in youth worldwide. In 2018, Damanhoury et al. collaborated with 46 international experts and established a consensus-based definition of MHO in the pediatric population, defining it as systolic and diastolic blood pressure ≤ 90th percentile, high-density lipoprotein cholesterol > 40 mg/dl (or > 1.03 mmol/l), triglycerides ≤ 150 mg/dl (or ≤ 1.7 mmol/l), and a measure of glycemia (4).

## Prevalence of MHO

In this issue, based on the modified NCEP criteria, Cai et al. observe that the prevalence of MHO in 15,114 Chinese young people aged 7-18 years, recruited in 2013, is 3.5% and is higher in boys (5.3%) than in girls (1.6%). Another cross-sectional study (Zin et al.) of 193 children with obesity aged 8-16 years from Malaysia, conducted in 2014, shows that the prevalence of MHO (the definition of which was also according to the modified NCEP criteria) is 30.1%. A further international collaborative study involving 3,497 young people aged 6-17 years from Brazil, China, Greece, Italy, and Spain suggests that the prevalence of MHO is 4.5% based on the NCEP criteria and 8.2% based on the IDF criteria (3).

## Associated factors of metabolic health status

One of the landmark studies determining the factors associated with metabolically healthy status concludes that the most important factors are low inflammatory markers, preserved insulin sensitivity, and less visceral adipose tissue compared to those classified as unhealthy (7). They additionally name lipid metabolism and atherosclerosis as potential related factors. Indeed, a recent position statement reinforces the idea that the location of adipose tissue (i.e., visceral and ectopic fat) impacts metabolic health status (8). Likewise, Zin et al. observe in children with obesity that those within the MHO phenotype had significantly lower body mass indexes, waist circumferences, and uric acid and higher adiponectin and apolipoprotein A-1 levels.

Interestingly, lifestyle factors such as physical activity and cardiorespiratory fitness are of great importance in characterizing a metabolic phenotype. In this regard, a systematic review and meta-analysis show that MHO individuals are more active, spend less time engaging in sedentary behaviors, and present higher levels of cardiorespiratory fitness than MUO (9). Specifically, among those studies involving children and adolescents, the evidence shows higher levels of moderate-to-vigorous physical activity, higher cardiorespiratory fitness levels, and a greater reduction in sedentary behaviors in MHO compared to MUO children (9). Furthermore, in this Research Topic, Viitasalo et al. show a gradual decrease in physical activity levels between childhood and adulthood in both metabolic phenotypes; the greatest reduction being in those classified as MUO.

Considering other factors, in this Research Topic, Cai et al. and Liu et al. report that after examining more than 12,000 children and adolescents from China, the most important risk factors associated to MHO in youth are: younger age; single-child status; urban residence; parental smoking; parental history of disease (i.e., obesity, hypertension, and diabetes); cesarean birth, premature birth, and birth following a prolonged delivery; breastfeeding for a prolonged duration; high birth weight; insufficient sleep; excessive screen time; and less physical activity.

Another aspect worth mentioning is the role of the metabolic phenotype in cardiac damage.

After studying a total of 459 children with obesity, Genovesi et al. conclude that the MUO phenotype might not be related to cardiac damage (i.e., left ventricular mass index and left ventricular hypertrophy).

## Conclusions

The current Research Topic further elucidates an important update on and new insights into metabolic phenotypes (healthy and unhealthy) in relation to obesity in children and adolescents. There is still a need to examine the transition from MHO to MUO through randomized clinical trials and to explore the physiological, genetic, and lifestyle factors that might be related to the development of an unhealthy metabolic phenotype and the reversal from an unhealthy to a healthy metabolic phenotype.

## Author contributions

BX and CC-S conceptualized, designed, wrote, and approved the Editorial. All authors contributed to the article and approved the submitted version.
